# Association of *MUTYH Gln324His *and *APEX1 Asp148Glu *with colorectal cancer and smoking in a Japanese population

**DOI:** 10.1186/1756-9966-27-49

**Published:** 2008-09-30

**Authors:** Mayumi Kasahara, Kayo Osawa, Kana Yoshida, Aiko Miyaishi, Yasunori Osawa, Natsuko Inoue, Akimitsu Tsutou, Yoshiki Tabuchi, Kenichi Tanaka, Masahiro Yamamoto, Etsuji Shimada, Juro Takahashi

**Affiliations:** 1 Faculty of Health Sciences, Kobe University Graduate School of Medicine, Kobe, Japan; 2 Osaka Cancer Immuno-Chemotherapy Center, Osaka, Japan; 3 Clinical Laboratory, Otemae Hospital, Osaka, Japan; 4 Department of Surgery, Yoshida Ardent Hospital, Kobe, Japan; 5 Department of Surgery, Yodogawa Christian Hospital, Osaka, Japan; 6 Department of Surgery, Kobe Rosai Hospital, Kobe, Japan; 7 Department of Surgery, Kobe Medical Center, Kobe, Japan

## Abstract

**Background:**

Genetic polymorphisms of DNA repair enzymes may lead to genetic instability and colorectal cancer carcinogenesis. Our objective was to measure the interactions between polymorphisms of repair genes and tobacco smoking in colorectal cancer.

**Methods:**

The case-control study involved sixty-eight colorectal cancer patients and 121 non-cancer controls divided into non-smokers and smokers according to pack-years of smoking. The genetic polymorphisms of DNA repair enzymes,*OGG1 Ser326Cys*, *MUTYH Gln324His*, *APEX1 Asp148Glu *and *XRCC1 Arg399Gln*, were examined using PCR-RFLP.

**Results:**

The *MUTYH Gln324His *showed strong significant associations with a risk of colorectal cancer (crude odds ratio [OR] 3.30, 95% confidence interval [95%CI] 1.44–7.60, p = 0.005; adjusted OR3.53, 95%CI 1.44–8.70, p = 0.006). The ORs for the *APEX1 Asp148Glu *were statistically significant (crude OR 2.69, 95%CI 1.45–4.99, p = 0.002; adjusted OR 2.33, 95%CI 1.21–4.48, p = 0.011). The ORs for the *MUTYH Gln324His *and the *APEX1 Asp148Glu *were statistically significant for colon cancer (adjusted OR 3.95, 95%CI 1.28–12.20, p = 0.017 for *MUTYH Gln324His *; adjusted OR 3.04, 95%CI 1.38–6.71, p = 0.006 for *APEX1 Asp148Glu*). The joint effect of tobacco exposure and the *MUTYH Gln324His *showed a significant association with colorectal cancer risk in non-smokers (adjusted OR 4.08, 95%CI 1.22–13.58, p = 0.022) and the *APEX1 Asp148Glu *was significantly increased in smokers (adjusted OR 5.02, 95%CI 1.80–13.99, p = 0.002). However, the distributions of *OGG1 Ser326Cys *and *XRCC1 Arg399Gln *were not associated with a colorectal cancer risk.

**Conclusion:**

Our findings suggest that the *MUTYH Gln324His *and the *APEX1 Asp148Glu *constitutes an increased risk of colorectal cancer, especially colon cancer. The *MUTYH Gln324His *is strongly associated with colorectal cancer susceptibility in never smoking history, whereas the *APEX1 Asp148Glu *genotype constitutes an increased risk of colorectal cancer when accompanied by smoking exposure.

## Introduction

Colorectal cancer is a major cause of death and is influenced by genetic characteristics and environmental factors. Humans are exposed daily to a large variety of toxic and carcinogenic compounds due to habits such as tobacco smoking. Tobacco smoking produces major classes of carcinogenic compounds: polycyclic aromatic hydrocarbons (PAHs), aromatic amines, and heterocyclic amines (HCA). Several of these compounds can produce bulky DNA adducts [1]. The colorectal mucosa is exposed to these compounds through either the alimentary tract or the circulatory system. DNA adducts were detected in the colonic mucosa of smokers than in nonsmokers [2]. A previous study found that heavy smokers have a 2–3-fold elevated risk of colorectal adenoma [3]. Our previous data showed that genetic polymorphisms of *NAT2 *and *CYP1A2 *in metabolic processes contributed to colorectal cancer risk depending on smoking status in Japanese population [4]. Therefore, tobacco smoking might be a potential risk factor for colorectal cancer.

DNA repair genes are increasingly being studied for cancer risk because of their critical role in maintaining genome integrity. The base excision repair (BER) pathway, one of four major DNA repair pathways, has a principal role in the repair of mutations caused by oxidized or reduced bases [5]. Therefore, polymorphisms of DNA repair genes may increase the risk of colorectal cancer. In addition, smoking-induced oxidative DNA base modifications and single-strand breaks are repaired by the BER pathway. In the current study, we focused on genes encoding four key proteins in the BER pathway: OGG1 (8-oxoguanine DNA glycosylase), MUTYH/MYH (Mut Y homolog), APEX1/APE1 (Apurinic/apyrimidinic endonuclease-1), and XRCC1 (X-ray cross-complementing group 1).OGG1 is a DNA glycosylase that removes 8-oxo-7, 8-dihydro-2'-deoxyguanosine (8-oxo-G), which is the most stable form of a highly mutagenic oxidative DNA adduct that pairs with cytosine [6]. MUTYH is another DNA glycosylase that removes adenine paired with 8-oxo-G or 1, 2-dihydro-2-oxoadenine (2-OH-A) paired with guanine [7]. The 2-OH-A level is increased by exposure to reactive oxygen species [8]. APEX1 removes abasic sites formed in DNA cleavage by OGG1 and MUTYH and recruits DNA polymerase β and DNA ligase III [9]. X-ray cross-complementing group 1 (XRCC1) is a multidomain protein that interacts with poly-ADP-ribose polymerase, DNA ligase III and DNA polymerase β, and repairs DNA single-strand breaks by generating a single nucleotide repair patch (short-patch BER)[10].

We conducted a hospital-based case-control study to elucidate the DNA repair gene polymorphisms, *OGG1 Ser326Cys *(rs1052133), *MUTYH Gln324His *(rs3219489),*APEX1 Asp148Glu *(rs1130409) and *XRCC1 Arg399Gln *(rs25487). *OGG1 Ser326Cys*, *APEX1 Asp148Glu *and *XRCC1 Arg399Gln *have also been linked to a risk of colorectal cancer [11-13]. Germ-line variants, *Tyr165Cys *and *Gly382Asp*, of the *MUTYH *gene have been associated with colorectal adenomas in Caucasians, not in Asians [14-16]. Recent studies reported that *MUTYH Gln324His *mutation was the most frequent mutation in Japanese patients with adenomatous popyposis, and the gene polymorphisms was associated with the risk of proximal colon cancer in the Japanese population [17,18]. To our knowledge, few previous studies have examined the effect of these polymorphisms on the association between smoking and colorectal cancer [19,20]. These polymorphisms were analyzed to evaluate genetic susceptibility to colorectal cancer and the possible modification effect on the relationship between smoking and colorectal cancer risk.

## Materials and methods

### Subjects

A total of 68 colorectal cancer patients (40 with colon cancer, 23 with rectal cancer, and 5 with unknown) were recruited into the study between October 2003 and March 2005 at the Kobe Medical Center and Kobe Rosai Hospital in Kobe, Japan. Patients were eligible if they underwent major colon or rectal surgery for a diagnosis of primary colon or rectal cancer. A total of 121 controls with no current or previous diagnosis of cancer were recruited between November 2002 and March 2003, as reported in our previous study [4]. The study design was approved by the Ethics Review Committee on Genetic and Genomic Research, Kobe University Graduate School of Medicine. Informed consent was obtained and detailed data on smoking were collected in a personal interview. The amount of smoke exposure was calculated in pack-years: the product of the number of years an individual had smoked and the average number of cigarettes smoked per day, converted into a standard pack of 20 cigarettes. Samples were coded after blood and data collection.

### Genotyping

**Figure 1:**
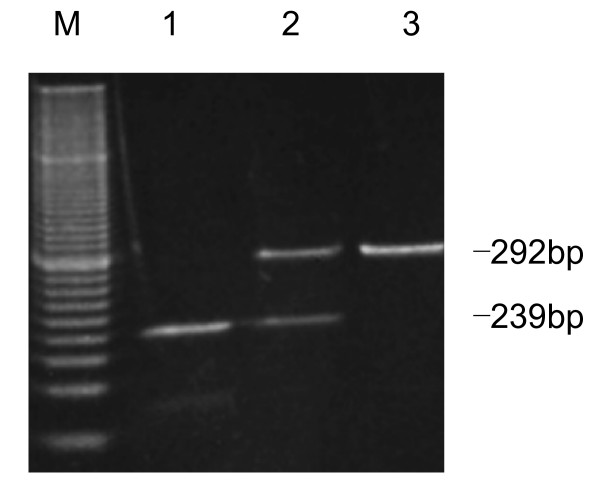
**Representative result for the *MUTYH Gln324His *polymorphisms by PCR-RFLP methods.** Lane M, markers; lane 1, His/His; lane 2, Gln/His; lane 3, Gln/Gln.

Genomic DNA used in the study was isolated for a previous study [4]. For *MUTYH Gln324His*, PCR was performed in a 25-μL reaction mixture containing 50 ng DNA, 250 mmol/l of each primer, 250 μmol/l of dNTPs, 1× PCR buffer, and 0.75 U of Ex *Taq *DNA polymerase (Takara, Shiga, Japan) using a programmable thermocycler PC-701 (Astec K.K, Fukuoka, Japan). The primer sequences were 5'-TGC CGA TTC CCT CCA TTC TCT CTT G-3' and 5'-TCT TGG CTT GAG TAG GGT TCG GG-3'. PCR conditions were 3 min at 94°C; 38 cycles of 1 min at 94°C, 1 min at 64°C, and 1 min at 72°C; followed by final extension for 10 min at 72°C. After amplification, the PCR products were digested with *HpyCH4 *III (New England Biolabs, Beverly, MA) for 4 h at 37°C in a final volume of 23 μl. Digested fragments were separated by electrophoresis on 12% polyacrylamide gel and visualized by ethidium bromide staining using a 20-bp DNA Ladder (Takara, Shiga, Japan) as a size marker. All experiments included positive and negative controls for each polymorphism. In PCR-RFLP genotyping of *MUTYH*, complete digestion of PCR products produced 292-bp fragments for the *Gln *allele, and 239-bp and 53-bp fragments for the *His *allele (the 53-bp fragment was too small to resolve accurately)(Figure 1). The genotypes of *OGG1 Ser326Cys *[21], *APEX1 Asp148Glu *[22], and *XRCC1 Arg399Gln *[23] were determined by PCR-RFLP analysis.

### Statistical analysis

Differences in demographic variables, smoking and grouped genotypic frequencies between the patients and controls were evaluated using a Chi-square test. All reported P-values are two-sided with p < 0.05 considered to be significant. Hardy-Weinberg equilibrium was tested using a Chi-square test to compare the observed genotype frequencies with the expected frequencies among the control subjects. The association between variant genotypes and risk of colorectal cancer was estimated by computing the odds ratios (OR) and the 95% confidence interval (95%CI) from unconditional logistic regression analysis, adjusted for age, gender, and smoking habit. Statistical analysis was performed with SPSS software (version 14.0 for Windows; SPSS Japan Inc., Tokyo, Japan). The subjects were divided into two groups according to pack-years of smoking: non-smokers (pack-years = 0), and smokers (pack-years > 0). The OR for each variant genotype was calculated for each subgroup.

## Results

The study included 68 patients and 121 controls (Table 1). The distribution of males (patients, 54.4%; controls, 61.2%) and females (patients, 38.2%; controls, 38.8%) did not differ significantly between the two groups (p = 0.872), and there was also no difference in the average age (± SD) of the patients (67.3 ± 10.9 years old) and controls (67.4 ± 6.7 years old) (p = 0.923). Non-smokers comprised 52.9% of patients and 45.5% of controls and smokers comprised 38.2% of patients and 49.6% of controls. There was no difference in smoking status between patients and controls (p = 0.253). Subsites were divided into 40 colon (58.8%) and 23 rectum (33.8%), and 5 unknown (7.4%).

**Table 1:** Characteristics of colorectal cancer case and control subjects

Item	Patients		Controls		*P-value*
			
	n	%	n	%	
Number	68		121		
Gender					
males	37	54.4	74	61.2	0.872^a^
females	26	38.2	47	38.8	
unknown	5	7.4	0	0.0	
					
Age					
~64	21	30.9	50	41.3	
65~69	13	19.1	29	24.0	
70~74	13	19.1	20	16.5	
75~	16	23.5	22	18.2	
unknown	5	7.4	0	0.0	
Mean ± S.D.	67.3 ± 10.9		67.4 ± 6.7		0.923^b^
					
Smoking status (Pack-years)					
non-smokers (Pack-years = 0)	36	52.9	55	45.5	0.253^a^
smokers (Pack-years > 0)	26	38.2	60	49.6	
unknown	6	8.8	6	5.0	
					
Subsites					
colon	40	58.8			
rectum	23	33.8			
unknown	5	7.4			

a: X^2 ^analysis

b: Student's T-test

Genotyping and allele frequencies of *OGG1 Ser326Cys*,*MUTYH Gln324His*,*APEX1 Asp148Glu*, and *XRCC1 Arg399Gln *adjusted for gender, age and smoking habit are shown in Table 2. The allele frequencies of the four gene polymorphisms in controls were consistent with the Hardy-Weinberg equilibrium. The ORs for the *OGG1 Ser/Cys *and *Cys/Cys *genotypes compared with the *Ser/Ser *genotype were not statistically significant (crude odds ratio [OR] 1.43, 95% confidence interval [95%CI] 0.73–2.78, p = 0.297; adjusted OR 1.43, 95%CI 0.69–2.95, p = 0.332). The ORs for the *MUTYH Gln/His *and *His/His *genotypes were shown to be statistically associated with the *Gln/Gln *genotype (crude OR 3.30, 95%CI 1.44–7.60, p = 0.005; adjusted OR3.53, 95%CI 1.44–8.70, p = 0.006). The ORs for the *APEX1 Asp/Glu *and *Glu/Glu *genotypes compared with *Asp/Asp *genotype were significantly increased (crude OR 2.69, 95%CI 1.45–4.99, p = 0.002; adjusted OR 2.33, 95%CI 1.21–4.48, p = 0.011). The ORs for the *XRCC1 Arg/Gln *and *Gln/Gln *genotypes compared with the *Arg/Arg *genotype were not statistically significant (crude OR 0.65, 95%CI 0.36–1.19, p = 0.164; adjusted OR 0.60, 95%CI 0.31–1.15, p = 0.125). These results indicate that the *MUTYH Gln324His *and the *APEX1 Asp148Glu *carry a significant risk for carcinogenesis of colorectal cancer.

**Table 2:** Genotype distribution in colorectal cancer and Allele frequency

									Allele frequency		
									
Genotype	patients (n = 68)		controls (n = 121)		crude		adjusted			patients	controls
							
	n	%	n	%	OR (95%CI)	*p*	OR (95%CI)^a^	*p*		%	%
*OGG1*											
Ser/Ser	17	25.0	39	32.2	1.00		1.00		Ser	52.9	54.6
Ser/Cys,Cys/Cys	51	75.0	82	67.8	1.43 (0.73–2.78)	0.297	1.43 (0.69–2.95)	0.332	Cys	47.1	45.5
*MUTYH*											
Gln/Gln	8	11.8	37	30.6	1.00		1.00		Gln	39.0	59.1
Gln/His,His/His	60	88.2	84	69.4	3.30 (1.44–7.60)	0.005	3.53 (1.44–8.70)	0.006	His	61.0	40.9
*APEX1*											
Asp/Asp	23	33.8	70	57.9	1.00		1.00		Asp	64.0	74.8
Asp/Glu,Glu/Glu	45	66.2	51	42.1	2.69 (1.45–4.99)	0.002	2.33 (1.21–4.48)	0.011	Glu	36.0	25.2
*XRCC1*											
Arg/Arg	42	61.8	62	51.2	1.00		1.00		Arg	78.7	71.1
Arg/Gln,Gln/Gln	26	38.2	59	48.8	0.65 (0.36–1.19)	0.164	0.60 (0.31–1.15)	0.125	Gln	21.3	28.9

a: OR adjusted for gender, age, smoking habit

The distributions of the four polymorphisms were compared among cases of colon and rectal cancer, and the OR adjusted for gender, age and smoking habit is shown in Table 3. The adjusted ORs for the *OGG1 Ser/Cys *and *Cys/Cys *genotypes compared with the *Ser/Ser *genotype were not statistically significant (OR 1.28, 95%CI 0.55–2.99, p = 0.567 for colon cancer; OR 1.66, 95%CI 0.56–4.87, p = 0.359 for rectal cancer). The adjusted ORs for the *MUTYH Gln/His *and *His/His *genotypes were significant compared with the *Gln/Gln *genotype for colon cancer, but not for rectal cancer (OR 3.95, 95%CI 1.28–12.20, p = 0.017 for colon cancer; OR 3.06, 95%CI 0.84–11.11, p = 0.089 for rectal cancer). The adjusted ORs for the *APEX1 Asp/Glu *and *Glu/Glu *genotypes compared with *Asp/Asp* genotype were statistically significant for colon cancer, but not for rectal cancer (OR 3.04, 95%CI 1.38–6.71, p = 0.006 for colon cancer; OR 1.61, 95%CI 0.64–4.09, p = 0.315 for rectal cancer). The adjusted ORs for the *XRCC1 Arg/Gln *and *Gln/Gln *genotypes compared with the *Arg/Arg *genotype were not statistically significant (OR 0.60, 95%CI 0.28–1.30, p = 0.194 for colon cancer; OR 0.62, 95%CI 0.24–1.58, p = 0.315 for rectal cancer).Therefore, the cancer subsite-specific study indicated that the *MUTYH Gln324His *and the *APEX1 Asp148Glu *have a colon cancer-specific risk.

**Table 3:** Genotype distribution in relation to subsites in colorectal cancer

Genotype	Colon cancer								Rectal cancer							
		
	patients (n = 40)		controls (n = 121)		crude		adjusted		patients (n = 23)		controls (n = 121)		crude		adjusted	
								
	n	%	n	%	OR (95%CI)	*p*	OR (95%CI)^a^	*P*	n	%	n	%	OR (95%CI)	*p*	OR (95%CI)^a^	*p*
*OGG1*																
Ser/Ser	10	25.0	39	32.2	1.00		1.00		5	21.7	39	32.2	1.00		1.00	
Ser/Cys,Cys/Cys	30	75.0	82	67.8	1.43 (0.63–3.21)	0.390	1.28 (0.55–2.99)	0.567	18	78.3	82	67.8	1.71 (0.59–4.95)	0.321	1.66 (0.56–4.87)	0.359
*MUTYH*																
Gln/Gln	4	10.0	37	30.6	1.00		1.00		3	13.0	37	30.6	1.00		1.00	
Gln/His,His/His	36	90.0	84	69.4	3.96 (1.32–11.95)	0.014	3.95 (1.28–12.20)	0.017	20	87.0	84	69.4	2.94 (0.82–10.49)	0.097	3.06 (0.84–11.11)	0.089
*APEX1*																
Asp/Asp	12	30.0	70	57.9	1.00		1.00		11	47.8	70	579	1.00		1.00	
Asp/Glu,Glu/Glu	28	70.0	51	42.1	3.20 (1.49–6.89)	0.003	3.04 (1.38–6.71)	0.006	12	52.2	51	42.1	1.50 (0.61–3.66)	0.376	1.61 (0.64–4.09)	0.315
*XRCC1*																
Arg/Arg	25	62.5	62	51.2	1.00		1.00		14	60.9	62	51.2	1.00		1.00	
Arg/Gln,Gln/Gln	15	37.5	59	48.8	0.63 (0.30–1.31)	0.217	0.60 (0.28–1.30)	0194	9	39.1	59	48.8	0.68 (0.27–1.68)	0.398	0.62 (0.24–1.58)	0.315

a: OR adjusted for gender, age, smoking habit

The ORs for the combined effect of tobacco exposure (pack-years) and the four polymorphisms, adjusted for gender and age, are shown in Table 4. The adjusted ORs for the *OGG1 Ser/Cys *and *Cys/Cys *genotypes compared with the *Ser/Ser *genotype showed no statistically significant risk in non-smokers and smokers (OR 1.14, 95%CI 0.41–3.13, p = 0.807 in non-smokers; OR 1.68, 95%CI 0.60–4.67, p = 0.321 in smokers). The adjusted ORs for the *MUTYH Gln/His *and *His/His *genotypes compared with the *Gln/Gln *genotype showed a significant association with colorectal cancer risk in non-smokers, but not in smokers (OR 4.08, 95%CI 1.22–13.58, p = 0.022 in non-smokers; OR 2.95, 95%CI 0.77–11.25, p = 0.113 for smokers). These results show that the *MUTYH Gln/His *and *His/His *genotypes are associated with colorectal cancer susceptibility with never smoking history. The adjusted ORs for the *APEX1 Asp/Glu *and *Glu/Glu *genotypes compared with the *Asp/Asp *genotype in smokers was significantly increased (OR 5.02, 95%CI 1.80–13.99, p = 0.002), whereas that in non-smokers did not show a significant (OR 1.56, 95%CI 0.66–3.68, p = 0.311). Smokers with the *APEX1 Asp/Glu *and *Glu/Glu *genotypes showed an increased risk of colorectal cancer. The adjusted ORs for the *XRCC1 Arg/Gln *and *Gln/Gln *genotypes compared with the *Arg/Arg *genotype were not statistically significant (OR 0.86, 95%CI 0.37–2.03, p = 0.732 in non-smokers; OR 0.43, 95%CI 0.16–1.16, p = 0.097 in smokers). These results indicate that the *MUTYH Gln324His* and the *APEX Asp148Glu* have statistically a significant risk of colorectal cancer according to smoking status.

**Table 4:** Genotype distribution in relation to smoking status in colorectal cancer

Genotype	non-smokers (Pack-years = 0)								smokers (Pack-years > 0)							
		
	patients (n = 36)		controls (n = 55)		crude		adjusted		Patients (n = 26)		controls (n = 60)		crude		adjusted	
								
	n	%	n	%	OR (95%CI)	*p*	OR (95%CI)^a^	*p*	N	%	n	%	OR (95%CI)	*p*	OR (95%CI)^a^	*p*
*OGG1*																
Ser/Ser	8	22.2	14	25.5	1.00		1.00		7	26.9	23	38.3	1.00		1.00	
Ser/Cys,Cys/Cys	28	77.8	41	74.5	1.20 (0.44–3.23)	0.725	1.14 (0.41–3.13)	0.807	19	73.1	37	61.7	1.69 (0.61–4.64)	0.310	1.68 (0.60–4.67)	0.321
*MUTYH*																
Gln/Gln	4	11.1	18	32.7	1.00		1.00		3	11.5	17	28.3	1.00		1.00	
Gln/His,His/His	32	88.9	37	67.3	3.89 (1.19–12.69)	0.024	4.08 (1.22–13.58)	0.022	23	88.5	43	71.7	3.03 (0.80–11.43)	0.102	2.95 (0.77–11.25)	0.113
*APEX1*																
Asp/Asp	15	41.7	29	52.7	1.00		1.00		8	30.8	41	68.3	1.00		1.00	
Asp/Glu,Glu/Glu	21	58.3	26	47.3	1.56 (0.67–3.65)	0.303	1.56 (0.66–3.68)	0.311	18	69.2	19	31.7	4.86 (1.80–13.13)	0.002	5.02 (1.80–13.99)	0.002
*XRCC1*																
Arg/Arg	20	55.6	29	52.7	1.00		1.00		18	69.2	30	50.0	1.00		1.00	
Arg/Gln,Gln/Gln	16	44.4	26	47.3	0.89 (0.38–2.08)	0.791	0.86 (0.37–2.03)	0.732	8	30.8	30	50.0	0.44 (0.17–1.18)	0.103	0.43 (0.16–1.16)	0.097

a: OR adjusted for gender, age

## Discussion

The association between the risk of colorectal cancer and polymorphisms of four DNA repair genes in the BER pathway was investigated in a small case-control study. No significant relationship was apparent between *OGG1 Ser326Cys *and colorectal cancer risk. Previous reports have suggested that *OGG1 Ser326Cys *is associated with colorectal cancer in Caucasians [11,24], but not among Koreans [25]. Our findings in a Japanese population are consistent with the results from the Korean population study.

Interestingly, we found that the *MUTYH Gln324His *genotype has a strong significant association with colorectal cancer risk, especially colon cancer. Tao *et al*. [18] reported *MUTYH Gln324His *in Japanese was statistically significantly associated with increased risk of proximal colon, but not distal colon or rectal cancer. Therefore, their results are consistent with our study. Moreover, a recent study found that the activity of *MUTYH Gln324His *is 34% less active than that of wild type [26]. 8-oxo-G is generated by direct oxidation of DNA by a hydroxyl radical, whereas 2-OH-A is exclusively generated by oxidation of dATP in the nucleotide pool [6,7]. The 2-OH-A level is increased in human cancerous tissues compared to normal tissues [27]. Thus, for colorectal cancer, it is also possible that the enzyme of *MUTYH Gln324His *may have partially impaired in repair of 2-OH-A opposite guanine, compared to repair of adenine opposite 8-oxo-G, because of the difference in the origin of each oxidized base. We also found that *MUTYH Gln324His *was statistically associated with increased risk in never smokers. These results suggest that the *MUTYH 324His *variation may be associated with a risk of colorectal cancer due to an increased mutation frequency, containing environmental factors except smoking.

We indicated that the *APEX1 Asp148Glu *genotype has a specifically association with colon cancer risk. A previous study reported that this genotype was especially an increased risk of colon cancer risk [28]. We also found a statistically significant association between the *APEX1 Asp148Glu *genotype and colorectal cancer risk in combination with smoking exposure. Ito et al. [29] reported that the gene-environment interaction between current smoking and *APEX1 148 Glu/Glu *genotype was statistically significant for lung cancer risk. However, a previous study didn't found about the effect of smoking habit on association between the *APEX1 Asp148Glu *genotype and colorectal cancer risk [28]. This polymorphism is located within the endonuclease domain of the protein [30], but it does not reduce endonuclease activity [31]. The 148 *Glu *allele has also been associated with increased mitotic delay after exposure to ionizing radiation [22]. Our results indicate that the *APEX1 *variation may play an important role in colorectal cancer risk, containing a reduced ability to communicate with the other BER proteins. In contrast, for *XRCC1 Arg399Gln *variants, we found no relationship with colorectal cancer. The *XRCC1 399Gln *allele has been linked with a reduced risk of colorectal adenomas [12,13], and XRCC1 has also been associated with improved progress in patients who underwent chemotherapy, but not in those who received surgery alone [11]. The smoking has an effect on colon adenoma risks among carriers of *XRCC1 *codon 399 *Arg *alleles [19,20]. However, we were unable to detect these relationships in our cases.

Our data may be biased by the relatively small number as a hospital-based case-control study, because we have several limitations. Therefore, we would require further verification as predictive biomarkers in a larger study population and need to clarify the gene-environment interaction between smoking and these genotypes.

In conclusion, *MUTYH Gln324His *and *APEX1 Asp148Glu *polymorphisms are important risk factors for colorectal cancer, especially colon cancer, in the Japanese population. In particular, the *MUTYH Gln324His *genotype is associated with colorectal cancer susceptibility in never smoking history, whereas the *APEX1 Asp148Glu *genotype constitutes an increased risk of colorectal cancer in combination with smoking exposure. *MUTYH Gln324His *and *APEX1 Asp148Glu *polymorphisms may be useful markers of genetic susceptibility to colorectal cancer.

## Competing interests

The authors declare that they have no competing interests.

## Authors' contributions

MK, KO and JT plan the study made all coordination and was involved in the laboratory processing. KY, AM, YO and NI participated in the study and performed the statistical analysis. AT, YT, KT, MY and ES carried out handling the samples. All authors read and approved the final version of manuscript.
